# Doxycycline post-exposure prophylaxis for prevention of sexually transmitted infections among Kenyan women using HIV pre-exposure prophylaxis: study protocol for an open-label randomized trial

**DOI:** 10.1186/s13063-022-06458-8

**Published:** 2022-06-16

**Authors:** Jenell Stewart, Elizabeth Bukusi, Fredericka A. Sesay, Kevin Oware, Deborah Donnell, Olusegun O. Soge, Connie Celum, Josephine Odoyo, Zachary A. Kwena, Caitlin W. Scoville, Lauren R. Violette, Susan Morrison, Jane Simoni, R. Scott McClelland, Ruanne Barnabas, Monica Gandhi, Jared M. Baeten

**Affiliations:** 1 Department of Global Health, University of Washington, Box 359931, 325 Ninth Ave, WA 98104 Seattle, USA; 2 Department of Medicine (Infectious Diseases), University of Washington, Seattle, USA; 3 Kenya Medical Research Institute (KEMRI), Kisumu, Kenya; 4 Department of Epidemiology, University of Washington, Seattle, USA; 5 Department of Biostatistics, University of Washington, Seattle, USA; 6 Department of Laboratory Medicine and Pathology, University of Washington, Seattle, USA; 7 Department of Psychology, University of Washington, Seattle, USA; 8 Department of Medicine, Division of HIV, Infectious Diseases, and Global Medicine, University of California San Francisco, San Francisco, USA

**Keywords:** STIs, Prevention, PrEP, Antibiotics, Women’s health, Doxycycline, Open-label, Randomized controlled trial

## Abstract

**Background:**

Women in Africa face disproportionate risk of human immunodeficiency virus (HIV) acquisition, accounting for more than half of new infections in Africa and similarly face a disproportionate burden of sexually transmitted infections (STIs). Very high STI prevalence is being observed globally, especially among people taking pre-exposure prophylaxis (PrEP) for HIV prevention. Doxycycline post-exposure prophylaxis (dPEP) has been proposed as an STI prevention strategy to reduce chlamydia, syphilis, and possibly gonorrhea, and trials are ongoing among cisgender men who have sex with men (MSM) and transgender women who are taking PrEP in high-income settings. We designed and describe here the first open-label trial to determine the effectiveness of dPEP to reduce STI incidence among cisgender women.

**Methods:**

We are conducting an open-label 1:1 randomized trial of dPEP versus standard of care (STI screening and treatment and risk-reduction counseling without dPEP) among 446 Kenyan women aged ≥ 18 and ≤ 30 years old women taking PrEP. Women are followed for 12 months, with quarterly STI testing, treatment, and adherence counseling. The primary trial outcome will be the combined incidence of *Chlamydia trachomatis*, *Neisseria gonorrhoeae*, and *Treponema pallidum*, compared between the randomized groups. We will also assess dPEP acceptability, tolerability, safety, impact on sexual behavior, adherence, and occurrence of antimicrobial resistance (AMR) in *N. gonorrhoeae* and *C. trachomatis* isolates. Finally, we will estimate cost per incident STI case and complications averted accounting for nonadherence and benefits relative AMR or side effects.

**Discussion:**

The results of this trial may have immediate implications for the global epidemic of STIs and sexual health. If effective, dPEP could put STI prevention into women’s hands. While dPEP may be able to prevent STIs, it carries important risks that could counter its benefits; global debate about the balance of these potential risks and benefits requires data to inform policy and implementation and our study aims to fill this gap.

**Trial registration:**

ClinicalTrials.gov NCT04050540.

## Administrative information

Note: the numbers in curly brackets in this protocol refer to SPIRIT checklist item numbers. The order of the items has been modified to group similar items (see http://www.equator-network.org/reporting-guidelines/spirit-2013-statement-defining-standard-protocol-items-for-clinical-trials/).

**Table:** 

Title {1}	Doxycycline post-exposure prophylaxis for prevention of sexually transmitted infections among Kenyan women using HIV pre-exposure prophylaxis: study protocol for an open-label randomized trial
Trial registration {2a and 2b}.	https://clinicaltrials.gov/ct2/show/NCT04050540
Protocol version {3}	Version 5.0, 30 September 2020
Funding {4}	US National Institutes of Health (grants R01AI145971, P30AI027757, K23MH124466)
Author details {5a}	Jenell Stewart,^1,2^ Elizabeth Bukusi,^1,7^ Fredericka A. Sesay,^1,3^ Kevin Oware,^7^ Deborah Donnell,^1,4^ Olusegun O. Soge,^1,2,5^ Connie Celum,^1,2,3^ Josephine Odoyo,^7^ Zachary A. Kwena,^7^ Caitlin W. Scoville,^1^ Lauren R. Violette,^2,3^ Susan Morrison,^1^ Jane Simoni,^6^ R. Scott McClelland,^1,2,3^ Ruanne Barnabas,^1,2,3^ Monica Gandhi,^8^ Jared M. Baeten,^1,2,3^. Departments of Global Health^1^, Medicine (Infectious Diseases)^2^, Epidemiology^3^, Biostatistics^4^, Laboratory Medicine and Pathology^5^, and Psychology^6^, University of Washington, Seattle, United States; Kenya Medical Research Institute (KEMRI)^7^, Kisumu, Kenya; Department of Medicine, Division of HIV, Infectious Diseases, and Global Medicine^8^, University of California San Francisco, United States.
Name and contact information for the trial sponsor {5b}	University of Washington Jared M. Baeten, MD, PhD 325 9^th^ Ave, Box 359,927, Seattle, WA, 98,104
Role of sponsor {5c}	The funder of the study had no role in study design, data collection, data analysis, data interpretation, or writing of the manuscript.

## Introduction

### Background and rationale {6a}

More than two million persons become newly infected with human immunodeficiency virus (HIV) each year, the majority in sub-Saharan Africa [1]. Cisgender women in Africa face disproportionate HIV risk, accounting for more than half of new infections, and with incidence rates that are often double or more than their male age-mates [2, 3]. At the same time, African women also face a disproportionate burden of sexually transmitted infections (STIs). Globally, the World Health Organization (WHO) estimates that 376 million new cases of four curable sexually transmitted infections—three bacterial pathogens (*Chlamydia trachomatis*, *Neisseria gonorrhoeae*, *Treponema pallidum* [etiologic cause of syphilis]), plus the protozoan parasite *Trichomonas vaginalis*—are acquired worldwide each year [4]. The consequences of bacterial STIs on sexual and reproductive health can be profound and lasting, e.g., pelvic inflammatory disease (PID), chronic pelvic pain, tubal infertility, pregnancy complications, fetal and neonatal death, and increased susceptibility to HIV [5–11], and are overwhelmingly borne by women.

The past decade has witnessed monumental strides in the development of highly-effective HIV prevention interventions, including antiretroviral pre-exposure prophylaxis (PrEP) [12–15]. PrEP reduces incident HIV but was not expected to prevent bacterial STIs, including gonorrhea, chlamydia, or syphilis. In high-income countries, like the US, the past decade has seen a resurgence in the incidence of bacterial STIs among men who have sex with men (MSM) [16, 17]. HIV and STIs overlap in transmission pathways through sexual exposure; thus, it is not surprising that high STI rates are seen among persons who use or are eligible to use PrEP. PrEP scale-up for eligible women is expanding rapidly. In 2017, Kenya was one of the first countries in the world to launch a national PrEP program, making PrEP available to all populations at risk for HIV, with a major focus on young women. PrEP roll-out provides an opportunity to improve HIV rates and target STI prevention interventions, in African women.

A recent open-label clinical trial among MSM in France (IPERGAY) found a 47% relative reduction in new bacterial STIs (specifically, either *C. trachomatis*, *N. gonorrhoeae*, or *T. pallidum*) among PrEP users who also took doxycycline following every sexual encounter [18]. This reduction was driven by reductions in incident *C. trachomatis* (70% reduction) and *T. pallidum*infections (73% reduction). The use of post-exposure doxycycline to prevent infections is already standard, recommended practice for other infectious diseases—for example, after tick exposure in areas of high Lyme disease prevalence or after flooding in leptospirosis endemic areas; doxycycline is also used as malaria pre- and post-exposure prophylaxis [19–21]. The concept of STI prophylaxis has a long history [18, 22, 23]. Several studies on single dose or monthly antibiotics among female sex workers in Asia and Africa, demonstrated reduced disease burden with mixed efficacy [23–29].

Conversely, multi-drug resistant *N. gonorrhoeae*is a growing international public health issue [30–35]. Notably, the IPERGAY results found no reduction in gonorrhea, which was expected given the high percentage (56%) of circulating strains of *N. gonorrhoeae*in Europe are already resistant to tetracyclines [36, 37]. While dPEP may prevent STIs, it carries important risks, particularly if intermittent doxycycline use induces tetracycline-resistant pathogens that could counter its benefits; global debate about the balance of these potential risks and benefits requires data to inform policy and implementation [38]. In Africa, antimicrobial resistance (AMR) data are sparse, but detection of plasmid-mediated tetracycline-resistant *N. gonorrhoeae*was highly prevalent (73–97%) [30, 31, 39, 40]. Rigorous studies are needed to quantify resistance in populations exposed to dPEP and whether dPEP use results in additional resistance.

Due to limited availability of etiologic STI testing in Africa, few studies have assessed STI risk among PrEP-using women in Africa. In those studies, STI rates are already comparable, with 27–53% *C. trachomatis*incidence per 100 person-years, to those seen among PrEP-using MSM in the US [41–43]. Prior to launching our pivotal trial, essentially all global conversation about dPEP for STI prevention has been directed towards MSM in high-income settings.

### Objectives {7}

Our clinical trial aims to determine the effectiveness benefit of dPEP to reduce STI incidence among Kenyan women taking PrEP for HIV prevention as well as assess associated risks of dPEP by exploring (a) safety, (b) acceptability, (c) adherence, and (d) resistance. We will also measure the cost of dPEP and estimate the cost per case averted, budget impact, and affordability.

### Trial design {8}

The dPEP Kenya study is a 1:1 open-labeled, randomized, superiority trial to determine the effectiveness benefit of dPEP to reduce STI (*N. gonorrhoeae, C. trachomatis, and T. pallidum* (syphilis)) incidence among women taking PrEP for HIV prevention.

## Methods: participants, interventions, and outcomes

### Study setting {9}

All study procedures will be conducted at a research clinic in Kisumu, Kenya.

### Eligibility criteria {10}

All participants are screened for eligibility and asked to provide oral and written informed consent before study participation. At screening, demographic information is obtained and participants are assessed for STI symptoms, HIV, and pregnancy. Prevalent STIs will be treated at enrollment. The results from the screening assessments are reviewed for the inclusion/exclusion criteria described below:

**Table:** 

Inclusion criteria	Exclusion criteria
1) Willing and able to give written informed consent 2) Age ≥ 18 years and ≤ 30 years old 3) Female sex at birth 4) HIV-seronegative, according to national HIV testing algorithm 5) Has a current prescription for PrEP, according to the national guidelines of Kenya	1) Pregnant^a^ 2) Breastfeeding a child^a^ 3) Allergy to tetracycline class 4) Current medications which may impact doxycycline metabolism or that are contraindicated with doxycycline, as per the prescribing information. These include systemic retinoids, barbiturates, carbamazepine, phenytoin, and warfarin 5) Recent use of prolonged (> 14-day course) antibiotics in the month prior to enrollment 6) Active, clinically significant medical or psychiatric conditions that would interfere with study participation, at the discretion of the site investigator or designee

^a^Use of doxycycline during pregnancy or breastfeeding historically discouraged, unless other drugs contraindicated or doxycycline is needed to treat severe illness, due to theoretical risk of cosmetic staining of primary teeth and possible effects on fetal bone development.

### Who will take informed consent? {26a}

All potential participants are screened for eligibility and then asked to provide oral and written informed consent by a trained, clinical study team member before study participation.

### Additional consent provisions for collection and use of participant data and biological specimens {26b}

In addition to consenting to participate in the study, participants opt in or out of agreeing to store samples at the Kisumu site and the University of Washington for future research into HIV, HIV- related diseases, STIs, and other infectious diseases.

## Interventions

### Explanation for the choice of comparators {6b}

We posit that there is equipoise to conduct a randomized evaluation at this time: while dPEP has demonstrated efficacy in one trial, it was relatively small in size and limited to MSM in France. Data specific to cisgender women are needed to know if dPEP is effective and safe. Additionally, the potential disadvantages of dPEP, such as antibiotic resistance, tolerability, and low adherence, have not been studied in women and justify the use of a control arm. Randomization in 1:1 allocation will be the most efficient for evaluating the efficacy of dPEP and balancing unknown risks and acceptability. The open-label design allows for optimal evaluation of effectiveness. First, the open-label model directly addresses questions about acceptability, as participants will react to dPEP itself, not a blinded, potentially placebo version. Second, an open-label design is likely to result in greater (and more realistic when implemented) adherence. Third, the design will directly allow assessment of theoretical sexual behavior changes (e.g., reduced stress) related to dPEP, which is only assessable if subjects know if they are actually taking active dPEP.

### Intervention description {11a}

Participants assigned to dPEP are instructed to take doxycycline hyclate 200 mg (two 100 mg capsules) orally within 24 h and up to 72 h after each vaginal receptive sex act as frequently as daily if indicated but not more than once daily.

### Criteria for discontinuing or modifying allocated interventions {11b}

Criteria for premature study treatment discontinuation include:

- Requirement for prohibited concomitant medications such as barbiturates, carbamazepine, phenytoin, methoxyflurane, acitretin, isotretinoin, and warfarin.
- Occurrence of an adverse event requiring discontinuation of doxycycline
- Request by the participant to terminate study treatment
- Clinical reasons believed life-threatening by the physician, even if not addressed in the toxicity section of the protocol
- Requirement for chronic tetracycline use (> 14 days)
- Pregnancy
- Breastfeeding

Participants who stop dPEP should be continued on study, off dPEP, with continued evaluations. In regard to pregnancy, participants will be counseled on current Kenyan guidelines, which do not recommend use of doxycycline in pregnant women and participants will stop dPEP should pregnancy occur. Women who are not using long-acting reversible contraception (LARC) will be screened for possible pregnancy at each quarterly visit using the WHO pregnancy checklist and, if needed, a urine pregnancy test will be conducted. Participants can resume dPEP when no longer pregnant or breastfeeding.

### Strategies to improve adherence to interventions {11c}

All participants receive 1:1 adherence counseling for PrEP with individualized barrier identification and problem-solving assistance as well as a check-in phone call within 1 week of enrollment to answer any questions about PrEP and/or dPEP when applicable. Participants in the dPEP arm receive 1:1 adherence counseling and covert, two-dose, pocket pill carriers, i.e., empty lipstick containers.

### Relevant concomitant care permitted or prohibited during the trial {11d}

Participants randomized to dPEP should report current and new medications to the study team to ensure no concern for drug interactions with doxycycline. The prescribing information for doxycycline hyclate should be reviewed to ensure no potential for drug interactions. Medications that interact with doxycycline include barbiturates, carbamazepine, phenytoin, methoxyflurane, acitretin, isotretinoin, and warfarin. Due to the time commitment from being in this study, participants may not be eligible to join this study if in other studies.

### Provisions for post-trial care {30}

Participants who are injured from participating in this study will be offered care at the study clinic, free of charge. No monetary compensation will be provided. If a participant requires medical care that the study clinic cannot provide, the study doctors will refer participants to the appropriate services or organizations that can provide care for the injury. If dPEP is shown to be efficacious, all study participants will be offered dPEP.

### Outcomes {12}

#### Primary study outcome STI testing

Combined incidence of *N. gonorrhoeae, C. trachomatis*, or early syphilis infection by laboratory-based diagnosis (e.g., positive *N. gonorrhoeae* or *C. trachomatis* nucleic acid amplification testing (NAAT) following a negative test or syphilis based first positive rapid plasma reagin (RPR) or fourfold increase in non-treponemal titers) as well as incidence of each pathogen individually every 3 months for 12 months.

#### Secondary outcomes

##### Safety

At each visit, participants in both study arms are asked about possible doxycycline side effects and symptoms, including non-specific symptoms which can occur in the absence of doxycycline.

##### Acceptability

This is explored using qualitative and quantitative data. Qualitative data collection includes semi-structured, in-depth individual interviews (*n* = 40) in the dPEP arm. Demographic and behavioral surveys are administered at each visit to investigate facilitators of and barriers to STI prevention and dPEP.

##### Adherence

A number of measures are used to determine adherence: Self-reported sexual behavior and, for those assigned to the dPEP arm, pill-taking behavior, are measured by timeline follow-back calendar [44] at each follow-up visit to assess exposures and dPEP use. Participants who consent to SMS surveys receive weekly SMS asking about the number of days in the last week during which they had sex. If a participant is in the dPEP arm, they will report the number of days in which dPEP was taken in a second SMS survey. Additionally, hair drug levels will be measured to assess quantitative use of doxycycline.

##### Resistance

All endocervical samples—baseline and follow-up, from both groups—with a positive NAAT result for *C. trachomatis* or *N. gonorrhoeae* will be tested for detection of the *tet*(C) cassette and plasmid-encoded *tet*(M) gene respectively, which is indicative of phenotypic resistance to tetracyclines, e.g., doxycycline [45].

##### Cost

We are estimating incremental costs, incurred and averted, of adding dPEP to PrEP.

### Participant timeline {13}

Enrollment began on February 5, 2020, and is expected to be completed in 18 to 24 months. Participants will be followed for 12 months each.

#### Study visits

**Fig. 1:**
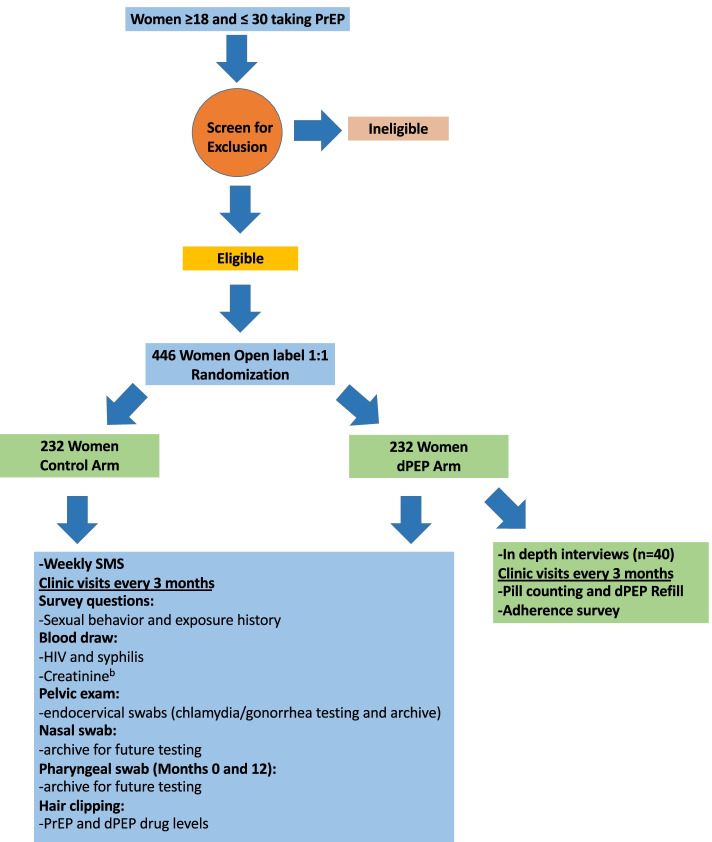
Flow diagram of participant follow-up procedures

Study visits are shown in Fig. 1.

#### Enrollment visit

Demographic and behavioral information are collected at enrollment. Pelvic exams for baseline STI testing, with sample storage, are performed and prevalent STIs are treated. Full medical review of symptoms and medications, blood testing (up to 20 mL of blood drawn), hair samples, and nasal and pharyngeal swabs are collected and pregnancy testing is done for all women at enrollment.

#### Quarterly visits after enrollment

At months 0, 3, 6, and 9, women randomized to dPEP receive doxycycline, sufficient for nearly daily use for 3 months. Unused capsules are counted and discarded at each follow-up visit (months 3/6/9/12). Doxycycline refills will be given to total 180 capsules for each 3-month period. The medication, doxycycline hyclate, is purchased from a quality-controlled supplier. Study visits, every 3 months, include questionnaires, risk reduction counseling, PrEP adherence counseling, a blood draw (up to 10 mL), HIV testing, a hair clipping, a nasal swab, a pharyngeal swab, rectal swabs (at the final visit only), WHO pregnancy checklist (with possible urine pregnancy test), and vaginal and endocervical swabs for etiologic STI testing. All participants diagnosed with an STI are treated the same day or return to the research clinic for treatment, using WHO/Kenya standard therapies (i.e., Ceftriaxone, Azithromycin, or Penicillin) avoiding doxycycline options, and a second visit two weeks following completion of STI treatment will be done for test of cure; this mitigates the potential risk to participants and the community if antimicrobial resistance is selected.

#### Unscheduled interim visits

Women may return to the study clinic for interim visits for any reason and will undergo STI testing and treatment if STI symptoms are present. Additionally, participants concerned about any adverse event from the study drug are invited to return for study provider evaluation and management.

#### Time and motion observations (length of quarterly visit)

A small random subset of participants in the dPEP group are asked to give verbal consent to have their study visit observed by a study team member to record time needed to provide this intervention (Table 1).

**Table 1:** Study procedures

Study visit month	*S*	0	3	6	9	12
Study coordination						
Obtain informed consent	X					
Screen for inclusion/exclusion	X	X				
Randomization		X				
Collect updated contact information	X	X	X	X	X	X
Reimbursement	X	X	X	X	X	X
Questionnaires						
Demographic information	X	X				
Behavioral questionnaire		X		X		X
HIV risk perception		X	X	X	X	X
Social Harms and Intimate partner violence (IPV)		X	X	X	X	X
Sexual behavior and exposure history		X	X	X	X	X
PrEP adherence		X	X	X	X	X
dPEP adherence (timeline follow-back calendar)			X ^a^	X ^a^	X ^a^	X ^a^
Fertility intention		X	X	X	X	X
Quality of Life		X	X	X	X	X
Clinical study care						
Medication review	X	X	X	X	X	X
General symptom assessment		X	X	X	X	X
WHO pregnancy checklist	X	X	X	X	X	X
STI symptom assessment	X	X	X	X	X	X
Supply condoms	X	X	X	X	X	X
Contraception counseling and provision/referral	X	X	X	X	X	X
Risk reduction counseling	X	X	X	X	X	X
dPEP pill count and refill			X^a^	X ^a^	X ^a^	X ^a^
Sample collection						
HIV testing (rapid test)	X		X	X	X	X
Urine pregnancy testing (rapid test)	X ^b^	X	X ^b^	X ^b^	X ^b^	X ^b^
Syphilis testing (serum rapid plasma reagin (RPR))		X	X	X	X	X
Creatinine (serum)		X^b^	X^b^	X^b^	X^b^	X^b^
CT/NG testing and resistance (endocervical swabs) ^c^		X	X	X	X	X
Hair PrEP and doxycycline drug levels		X	X	X	X	X
Trichomonas screening (vaginal swab)		X	X	X	X	X

*S* screening visit

^a^intervention arm only

^b^if clinically indicated

^c^Participants diagnosed with an STI will return 2 weeks following treatment for test of cure

### Sample size {14}

The sample size is based on anticipated combined STI incidence of 22% per year in the standard of care arm, which is less than the *C. trachomatis*incidence alone seen in recent studies [41–43]. Calculation is based on time to first incident STI alone, although we anticipate that some women will have repeat STIs contributing additional events and additional statistical power. We have the power to detect a 50% effect, comparable to the IPERGAY results, and of sufficient benefit to have important public health implications. We anticipated that the effect of *C. trachomatis* could be strong (> 50%) given universal susceptibility to doxycycline and IPERGAY findings. To achieve 80% power to detect a 50% reduction in women experiencing any STI, a total of 66 events is required: assuming a standard of care incidence of 22% and 10% loss to follow-up, using a two-sided alpha of 0.05, a sample size of *n* = 446 is needed (*n* = 223 per arm). The trial intends to have sufficient power to detect 50% effectiveness in *C. trachomatis* alone (predicted to be more than the minimum number of composite events for the endpoint of any STI).

### Recruitment {15}

We will be recruiting from clinics providing PrEP in Kisumu, Kenya. The experienced community outreach team at the study site in Kisumu employs community-based mobilization strategies, including engaging community health volunteers, gate keepers, peer-to-peer mobilization, youth peer providers and ambassador models, use of printed and electronic IEC materials, radio talk shows in English, *Kiswahili*, and the local *Dholuo* dialects, active participation in local social events for information sharing, and partnering with learning institutions to give health talks and to provide study-related information. Careful attention to confidentiality is made in the recruitment process and potential participants are never approached individually in group settings or public messaging. Recruitment messaging is screened for cultural and community acceptability to reduce risk of stigma for study participants. Potential participants who are not interested in the study or do not meet study eligibility criteria may be notified of alternative studies that they may be eligible for or provided information about where to access standard of care for HIV prevention and STI services.

## Assignment of interventions: allocation

### Sequence generation {16a}

Participants will be randomized 1:1 to dPEP versus standard of care; randomization will be done in variable-sized blocks and using a computer-based randomization system, Randomize.net, following consent and eligibility confirmation. A limited number of envelopes (*n* = 18) are available at the research site for use in randomization allocation in the case of power or internet outage and the site is therefore unable to access Randomize.net using a computer or a mobile phone.

### Concealment mechanism {16b}

Blinded study assignment, or, when necessary, opaque, back-up envelope system assignment, is implemented via http://www.randomize.net.

### Implementation {16c}

Unblinded members of the statistical team at the University of Washington (UW) generate the allocation order for randomization and provided separate allocation lists to Randomize.net, as well as lists of valid participant IDs. Copies of the code used to generate the randomization, and the randomization list itself, is stored in a secure directory, to which only unblinded members of the UW dPEP Kenya statistical team have access.

The randomization sequence is implemented in sequential order. Site staff enter a participant ID on Randomize.net, the participant ID is verified through Randomize.net, and the next sequential treatment arm is assigned. The date of randomization is reported on the study case report forms.

## Assignment of interventions: blinding

### Who will be blinded {17a}

In this open-label trial, blinding is limited to laboratory staff, the endpoint adjudication committee, principal investigators, and project director.

### Procedure for unblinding if needed {17b}

Not applicable for open-label trial.

## Data collection and management

### Plans for assessment and collection of outcomes {18a}

#### Questionnaires and interviews

##### Quantitative data

Demographic and behavioral surveys are administered at each visit, using questions we have used in our prior trials pertaining to socioeconomic status, demographic factors, alcohol use, depression, HIV risk perception, prevention strategies, social harms, relationship, sexual behavior, fertility intention, and HIV stigma.

##### Qualitative work

Qualitative data collection includes semi-structured in-depth individual interviews (IDI) (*n*= 40) in the dPEP arm [46]. The serial IDI is conducted soon after enrollment to get information on early experiences and at Months 6 and 12 after participants have experienced dPEP. Interviews are conducted using the participants’ preferred language (English, *Dholuo*, or *Kiswahili*). Participants are sampled for the qualitative interviews to include diversity in relationship status, adherence level, and participation in transactional sex (stratified-purposive sampling).

#### Outcome measures

##### Primary study outcome STI testing

A provider-collected endocervical swab is used to test for *N. gonorrhoeae*/*C. trachomatis* and blood sample is used for syphilis serology testing. Rapid plasma reagin (RPR) is conducted at least 30 days after last syphilis treatment and must be interpreted in the context of prior test results and current symptoms and exposure history. Testing is conducted by staff blinded to randomization assignment, and STIs are reviewed by an Endpoint Adjudication Committee blinded to the treatment arm.

##### Secondary outcomes

*Safety*: At each visit, participants in both study arms are asked about possible doxycycline side effects, including esophagitis, rash, gastrointestinal symptoms, photosensitivity, headache, and vaginal yeast infection. Control arm participants are asked about these symptoms to establish a comparator for non-specific symptoms which can occur in the absence of doxycycline. Discontinuations (in the dPEP arm only) are also measured and questionnaires assessing pregnancy, social harms and sexual behavior are administered at each study visit. In addition, all adverse events (AEs) that are attributed to doxycycline in the opinion of the site investigator or meet serious adverse events (SAEs) definition are recorded on data collection forms.

*Acceptability*: This is explored using qualitative and quantitative data. *Qualitative work:* Qualitative data collection includes semi-structured in-depth individual interviews based on the theoretical framework of acceptability. Participants are sampled for the qualitative interviews to include diversity in relationship status, adherence level, and participation in transactional sex (stratified-purposive sampling). *Quantitative data:* Demographic and behavioral surveys are administered at each visit to investigate facilitators of and barriers to STI prevention and dPEP. These surveys capture data on socio-economic status, demographic factors, alcohol use, depression, social harms, HIV stigma, HIV risk perception etc. Longitudinal change in qualitative themes and associated barriers and facilitators will be assessed and analyzed.

*Adherence*: All participants receive 1:1 adherence counseling for HIV PrEP with individualized barrier identification and problem-solving assistance. Participants in the dPEP arm receive 1:1 adherence counseling as well as reminders on their daily PrEP tablet bottle as a reminder to assess personal need for dPEP on that day [47, 48]. A number of measures are used to determine adherence: *Self-reported measures:* Sexual behavior and, for those assigned to the dPEP arm, pill-taking behavior, are measured by timeline follow-back calendar [44] at each follow-up visit to assess exposures and dPEP use. Using a timeline follow-back calendar, participants will be asked when dPEP was taken and if each sex act was covered by dPEP in the past 2 weeks. *Real-time behavioral measurement:* Participants who consent to SMS receive weekly SMS sent in English, *Kiswahili*, or *DhoLuo* as part of a momentary ecological assessment of adherence and exposure. Exposure, i.e., have penetrative sex, is referred to by a code word for added privacy, and all participants receive the same weekly text asking about the number of days in the last week during which they had a potential exposure. If a participant is in the dPEP arm, they will receive a second SMS, “How many times did you take dPEP this week?” Encrypted transmission, password protection to open the surveys, and immediate deletion permit sensitive data to be collected safely. *Objective measures:* Hair drug levels of doxycycline are measured to assess quantitative exposures more objectively to dPEP, for several analyses. Hair samples (50-100 strands) are collected following consent to collection and confirmed eligibility for hair collection (occipital hair >0.5cm in length with absence of bleaching) from all participants at each visit (Months 0, 3, 6, 9, and 12).

*Resistance*: All endocervical samples – baseline and follow-up, from both arms – with a corresponding positive NAAT result for *C. trachomatis* and/or *N. gonorrhoeae*, will be stored at -80C and then tested for detection of the *tet*(C) cassette in *C. trachomatis*, the plasmid-encoded *tet*(M) gene in *N. gonorrhoeae*and/or genetic resistance determinants which is indicative of phenotypic resistance to tetracycline, doxycycline, and minocycline [45].

*Cost*: We will estimate incremental costs, incurred and averted, of adding dPEP to PrEP. Activity-based micro-costing studies will be conducted to estimate costs incurred (e.g., start-up activities, training, and dPEP) and costs averted (diagnosis and treatment of symptomatic STIs, infertility, and adverse pregnancy outcomes). Also, cost data will be collected from the study budget, published reports, and the literature. Time and motion studies will be conducted by observing visits and staff time spent on dPEP initiation and adherence counseling, and clinical procedures. The total time required for the intervention will be estimated, adjusting for time spent on research activities. Using these data, the time and costs for dPEP will be estimated. We will adapt compartmental, dynamic models to simulate STI transmission in Kisumu.

### Plans to promote participant retention and complete follow-up {18b}

Visits will take place at enrollment and quarterly thereafter, up to 12 months; we will conduct tracing in the case of missed visits to ensure high retention. Flexible clinic scheduling is available to promote high rates of follow-up. We will make efforts to retain all subjects at their final visit to meet standard of care in addition to ensuring complete information is available for the entire population. We will follow standard Kenyan clinical practices for notifying participants of their study follow-up visits, which include sending participants reminders using SMS messaging and telephone calls.

### Data management {19}

REDCap electronic data capture tools are hosted at the Institute of Translational Health Sciences, University of Washington. REDCap (Research Electronic Data Capture) is a free, secure, web-based application designed to support data capture for research studies, providing (1) an intuitive interface for validated data entry; (2) audit trails for tracking data manipulation and export procedures; (3) automated export procedures for seamless data downloads to common statistical packages; and (4) procedures for importing data from external sources. The REDCap database is only accessible to approved users and permissions to export data are limited to the data management team.

Data logic checks, ranges for data values, and requirements to avoid missing data are built into the REDCap database. The site data management staff reviews each CRF for completion and locks the record to avoid accidental data changes. Data quality control checks are conducted by the site data entry staff and by the UW data manager on a rolling basis and on all data at least quarterly. Cumulative data exports are downloaded from REDCap each week by the UW data manager and are stored on an encrypted drive on a password-protected computer.

### Confidentiality {27}

Every effort will be made to protect participant privacy and confidentiality to the extent possible. Personal identifying information will be retained at the Kisumu study site and not forwarded to the labs in Mombasa, San Francisco, or Seattle, or to the University of Washington team; instead, all information will be identified only by study ID number. The site will use their standard operating procedure for confidentiality protection that reflects the input of study staff and community representatives to identify potential confidentiality issues and strategies to address them. All study-related information will be stored securely at the study sites. All participant information will be stored in areas with limited access. Data collection, administrative forms, laboratory specimens, and other reports will be identified only by a coded number to maintain participant confidentiality. All records that contain names or other personal identifiers, such as locator forms and informed consent forms, will be stored separately from study records identified by code number. All local databases will be secured with password-protected access systems. Forms, lists, logbooks, appointment books, and any other listings that link participant ID numbers to other identifying information will be stored in a separate, locked file in an area with limited access.

### Plans for collection, laboratory evaluation, and storage of biological specimens for genetic or molecular analysis in this trial/future use {33}

A pelvic exam will be performed at enrollment and all quarterly study visits for all participants. Participants will be encouraged to coordinate study visits prior to or following menstruation.

All visits will include blood sample collection for syphilis serology testing (incident infection = fourfold rise in non-treponemal RPR titer). RPR should be conducted at least 30 days after last syphilis treatment and must be interpreted in the context of prior test results and current symptoms and exposure history. As above, hair samples will be collected to measure cumulative doxycycline drug-taking.

We will archive (at − 80 °C) nasal swabs, pharyngeal swabs, vaginal swabs, endocervical swabs, rectal swabs, and serum samples for potential future testing, including for studies of commensal *Staphylococcus aureus* and other organisms detection and resistance, bacterial vaginosis, vaginal microbiome, semen exposure, *Mycoplasma genitalium*, rectal *C. trachomatis* infection or colonization and microbiome resistance patterns.

Samples will be tested within Kenya where feasible. Any samples that require specialized testing will be shipped to the University of Washington.

All STI screening will be completed with point-of-care testing in Kisumu, Kenya (Cepheid GeneXpert) or in Mombasa, Kenya (Aptima Combo 2). Endocervical swabs will be tested for *C. trachomatis* and *N. gonorrhoeae* by nucleic acid amplification testing (NAAT), and vaginal swabs will be tested for *Trichomonas vaginalis* by NAAT.

Hair samples from all participants will be collected, but only a subset will be shipped from the University of Washington to the University of California San Francisco Hair Analytical Laboratory (HAL), which uniquely is able to perform the required testing. Samples from three groups of participants will be analyzed: (1) 50 participants in the dPEP arm for dPEP and PrEP drug levels at each follow-up visit (months 3, 6, 9, and 12), (2) any dPEP arm participant with incident STI, and (3) random sample of 10% all-comers (*n* = 50) at enrollment (month 0) and 5% of control arm at follow up visits. Samples will be analyzed using liquid chromatography-tandem mass spectrometry (LC–MS/MS) to measure doxycycline hair level exposure over time (hair segments represent weekly exposures); hair levels of TDF and FTC are a long-term metric of exposure to PrEP. Testing of additional samples may be done if the results of this pre-defined sample warrant.

Endocervical samples—baseline and follow-up, from both arms—with a positive NAAT result for *C. trachomatis* and/or *N. gonorrhoeae*, will be shipped to the University of Washington laboratory of Dr. Soge for molecular testing. DNA will be purified from residual endocervical NAAT samples and endocervical swabs using the *High Pure* DNA *Kit* (Roche Diagnostics, Indianapolis, IN). The impact of dPEP on *C. trachomatis* and *N. gonorrhoeae* resistance will be measured by comparing the overall rates of doxycycline-resistant events occurring in the dPEP arm to rates in the control arm, at baseline and separately during follow-up adjusted for medical factors that confound the dPEP-resistance risk relationship.

Molecular characterization of *N. gonorrhoeae* will include strain typing using the internationally frequently used *N. gonorrhoeae*multiantigen sequencing typing (NG-MAST) system [49]; and detection of *tet*R including plasmid-mediated *tet*(M); and chromosomally-mediated resistance determinants: mtrR (A39T, G45D, G45S, − 35delA and − 10insTT), rpsJ (V57M), porB (G120K, A121D/N), and pilQ (E666K) by PCR and sequencing [50]. Well-characterized positive control strains and negative controls will be included in all PCR assays [45]. The detection of the plasmid-encoded *tet*(M) gene and/or *tet*R genetic resistance determinants is indicative of phenotypic resistance to tetracycline, doxycycline, and minocycline [45]. All samples with the same NG-MAST sequence types will be further differentiated by multilocus sequence typing. Whole genome sequencing will be conducted on endocervical swabs from patients with the same strain types and *tet*R genotypes and rigorous investigation of single nucleotide polymorphisms associated with novel resistance mechanisms will be performed [51].

The horizontally acquired genomic island which *encodes* the tetracycline efflux pump *tet*(C), found in *Chlamydia suis*strains from pig [52], is the only reported *tet*R determinant in the genus *Chlamydia *[53]. Therefore, we will screen for the presence of *tet*(C) in all samples found to be positive for *C. trachomatis;* and use real-time PCR assays for genotyping of *C. trachomatis* including *Lymphogranuloma venereum* (LGV) associated L-serovars L1, L2, or L3.

## Statistical methods

### Statistical methods for primary and secondary outcomes {20a}

Comparison of STI incidence by arm will be conducted by estimating the relative risks of any STI over the months 3, 6, 9, and 12 visits using a modified Poisson model fitted using GEE methods to account for repeated observations from each participant, assuming an independent covariance structure, with study arm as the only covariate. The test for significance will be a two-sided alpha = 0.05. 95% confidence intervals will be computed using robust standard errors. The same analysis will be repeated for the individual STIs.

Assessment of difference in odds of tetracycline resistance by arm across all post baseline visits with infection (*N. gonorrhoeae* and *C. trachomatis*, separately) will be assessed by estimating the odds of tetracycline resistance using logistic regression, with repeated measures methods (GEE) if repeat infections occur. Additionally, symptoms, collected at each visit, will be tabulated for each arm to evaluate the tolerability of dPEP. SMS surveys collected weekly ask all participants how many days of the last week they had sexual intercourse with responses ranging from 0–7. We will compare the frequency of sexual activity over time to investigate whether they differ by arm using a GEE with the Poisson distribution.

To evaluate adherence, a tabulation of # days sex and # days taking dPEP will be calculated for each measure to describe the distribution of coverage proportion for each woman, overall follow-up, by quarterly visit. A trial statistical analysis plan will be finalized prior to unblinding of results and will be made available with the primary trial report.

### Interim analyses {21b}

The trial will be monitored by an independent data monitoring committee approximately every 6 months. The trial plans to continue until we have established definitive efficacy results, whether effective or ineffective, for both *C. trachomatis* alone and for any STI. Two formal interim monitoring times are planned, when 1/3 and 2/3 of the follow-up visits have been completed, with stopping rules based on consideration of both the primary outcome and the *C. trachomatis* outcome alone. Because the outcomes are highly correlated, no adjustment for multiple comparisons is planned. O’Brien-Fleming boundaries will be used, with stopping based on the alpha spending corresponding to observed p-values in the efficacy analyses. Stopping for both efficacy and lack of efficacy will be considered; however, a decision to stop for efficacy will need to balance the need for evidence assessing the potential harm of acquired tetracycline resistance.

### Methods for additional analyses (e.g., subgroup analyses) {20b}

Several sensitivity analyses are planned:

1. Time to first STI using Kaplan–Meier and Cox PH regression
2. Including STIs diagnosed at test of cure visits (intended to include infections that are not successfully cured with the initial treatment). The primary outcome will be modified to include any STI re-diagnosed within 28 days of the scheduled quarterly visits to account for missed incident infections in conservative exclusion in 28-day period when lingering molecular components of pathogens can trigger a positive STI screen despite successfully treated infection, and the statistical analysis for the primary analysis repeated.
3. Analysis counting all individual STIs diagnosed in a quarterly interval. A Poisson model fitted using GEE methods to account for multiple events from each participant will be used to estimate the change in rate of infections between arms. If necessary to achieve a better fit, a zero-inflated Poisson model may be used.

### Methods in analysis to handle protocol non-adherence and any statistical methods to handle missing data {20c}

A per-protocol analysis is planned where participants assigned to the dPEP arm will be restricted to study time prior to the first discontinuation of study drug. The evaluation of the impact of doxycycline on any STI will use the same model as specified for the primary analysis, but also potentially adjusted for age, baseline STI, baseline and follow-up sexual behavior (number of partners, nulliparous, contraceptive use).

### Plans to give access to the full protocol, participant-level data, and statistical code {31c}

The final study protocol will be available upon publication of the primary results. Deidentified, individual participant data, and statistical code aligned with primary results will be made available in agreement with overseeing ethical review committees.

## Oversight and monitoring

### Composition of the coordinating center and trial steering committee {5d}

The entire project will be jointly led by the University of Washington International Clinical Research Center and Kenya Medical Research Institute- Kisumu to jointly manage the coordination of site and laboratory-based activities.

### Composition of the data monitoring committee, its role, and reporting structure {21a}

An independent external Data Safety and Monitoring Board (DSMB) will be established to protect participant.

safety by monitoring study outcomes, implementation, and data quality. The board will consist of expert clinicians, statisticians, and scientists, including Kenyans, in the field of STI treatment and prevention, antimicrobial resistance, and PrEP delivery in sub-Saharan African settings. The DSMB membership will be restricted to individuals free of apparent significant conflicts of interest. The source of these conflicts may be financial, scientific, or regulatory in nature.

The DSMB will be responsible for the review of operational, endpoint, and other data, including unblinded data, for a purpose of making recommendations to the trial leadership team regarding the safety and interests of trial participants. The DSMB will assess the relative safety and relative effects of the intervention during the trial and will monitor the overall conduct of the clinical trial. The DSMB will meet via teleconference on a schedule defined prior to study initiation (generally, prior to the trial start and then every 6 months, but timing will depend on the accumulation rate of trial endpoints). The DSMB will be charged with the decision to recommend stopping the study early if interim analyses demonstrate emergent differences between the trial arms meeting pre-defined levels of significance, significant change in rate of doxycycline-resistant STIs, or significant safety concerns. At a minimum, a DSMB meeting must have a quorum of the chair and 2 other members. One or more Formal Interim Analysis meetings will be held to review data relating to relative effects of treatment on the trial outcomes.

Patient safety and quality of trial conduct will also be reviewed at these meetings. The study team will carefully document any participants who become excluded from intervention arm due to pregnancy or are breastfeeding for careful review by DSMB for any adverse events to pregnant women or infants. Formal interim monitoring guidelines will be discussed at the Organizational Meeting prior to trial initiation.

To contribute to enhancing the integrity of the trial, the DSMB may also formulate recommendations relating to the selection/recruitment/retention of participants, their management, improving adherence to protocol-specified regimens, retention of participants, and the procedures for data management and quality control. The study leadership will be responsible to promptly review the DSMB recommendations, to decide whether to continue or terminate the trial, and to determine whether amendments to the protocol or changes in study conduct are required. The leadership is jointly responsible with the DSMB for safeguarding the interests of participating patients and for the conduct of the trial. The DSMB will be notified of all changes to the protocol or to study conduct. The DSMB concurrence will be sought on all substantive recommendations or changes to the protocol or study conduct prior to their implementation. In addition, the DSMB will provide guidance about results from other studies of dPEP that may be ongoing current with this trial. Reports from all reviews will be provided for submission to overseeing IRBs/ECs.

### Adverse event reporting and harms {22}

The study team will monitor the conduct of the study in real-time through monthly summary reports of arms of accrual, and baseline characteristics and quarterly reports of data pooled over treatment arms of data completeness, specimen collection, and adverse events (AEs). The study team will review individual participant-level safety data frequently to assess the relation of all reported AEs to study treatment. On a monthly basis, the study team will review summaries of premature study discontinuations and premature study treatment discontinuations (and reasons) and AEs. All AEs that are recorded must have their severity graded. To grade AEs, sites should refer to the most recent version of the Division of AIDS Table for Grading the Severity of Adult and Pediatric Adverse Events (DAIDS AE Grading Table).

### Frequency and plans for auditing trial conduct {23}

#### Early study assessment

The first assessment should occur within three months of the first enrollments.

#### Routine interim assessments

Typically, assessments occur approximately every 4–6 months and include review of operations and management of site, regulatory compliance, informed consent and eligibility, safety, CRF data, and source document quality, compliance with following of protocol, EQA, review of laboratory systems and study drug product control. Intervals may be longer or shorter than stated above, dependent on participant enrollment rate, quality issues, study compliance, or other study or site issues. Options for virtual monitoring or partial/targeted monitoring will be permitted due to restrictions on travel and staffing during the COVID-19 pandemic.

#### For cause assessments

If there are specific concerns, complaints, or additional investigation is required, ad hoc assessments may be conducted.

### Plans for communicating important protocol amendments to relevant parties (e.g., trial participants, ethical committees) {25}

The study protocol, site-specific informed consent forms, participant education, and recruitment materials, and other requested documents—including any subsequent modifications—will be reviewed and approved by the UW IRB, KEMRI Scientific Ethics Review Unit (SERU), and Pharmacy and Poison Board (PPB) Expert Committee on Clinical Trials (ECCT) in accordance with their requirements; they will be responsible for oversight of research conducted at the study site. Subsequent to the initial review and approval, the UW IRB will review the study at least annually.

### Dissemination plans {31a}

The investigators are committed to sharing data and other unique resources from this project with qualified individuals in the scientific community. The University of Washington International Clinical Research Center has a Manuscripts and Ancillary Studies Committee established to enable data and specimen collaborative work from studies conducted through the research group. The International Clinical Research Center routinely shares data, biologic specimens, laboratory data, project materials, training manuals, data collection forms, qualitative interview guides, and other resources from studies through this mechanism, from its many completed and ongoing studies. Intellectual property and data generated under the proposed project will be administered in accordance with both University of Washington and NIH policies, including the NIH Data Sharing Policy and Implementation Guidance of March 5, 2003. Materials generated under the project will be disseminated in accordance with University/Participating institutional and NIH policies. Depending on such policies, materials may be transferred to others under the terms of a material transfer agreement. The International Clinical Research Center has efficient mechanisms in place to complete such agreements and transfer data, specimens, and other materials, while abiding by all regulatory, ethical, and confidentiality requirements. Access to databases generated under the project will be available for educational, research, and non-profit purposes as approved by the relevant IRBs. Publication of data shall occur during the project, if appropriate, or at the end of the project, consistent with normal scientific practices. We will publish our findings in a timely fashion and will present unpublished data at appropriate research conferences. We are committed to collaboration in complex disease research and would participate in data-pooling studies as allowed by our currently established consent forms and IRBs.

## Discussion

PrEP roll-out in several African countries, ongoing and upcoming, provide an opportunity to impact both HIV and STI rates in women with the addition of STI-focused programming. The consequences of cervical/uterine STIs are potentially severe and STI prevention is a priority for women in PrEP trials who reported access to STI services as a prime motivation for their participation [54, 55]. The current standard of care for STI control in most resource-limited settings is syndromic management, which has very poor sensitivity (< 20%) for vaginal and cervical STIs compared to etiologic testing [56], and low positive predictive value (approx. 50%) [57, 58]. Giving doxycycline post-exposure prophylaxis, or dPEP, could be an inexpensive intervention, especially in settings where testing is unavailable or unaffordable. In contrast to other STI prevention strategies like male condoms and partner notification which rely on partner participation, dPEP would be woman-controlled.

This is the first trial of dPEP for STI prevention to enroll cisgender women. This population disproportionately bears the global burden of morbidity and mortality from STIs. Data specific to women are needed to know if dPEP is effective and safe for this large, priority population. Additionally, the potential disadvantages of dPEP, such as antibiotic resistance, tolerability, and low adherence, have not been studied in women and justify the use of a control arm while balancing unknown acceptability with a 1:1 randomization. We are using an open-label design to allow for optimal evaluation of effectiveness. First, the open-label model directly addresses questions about acceptability, as participants will report acceptability and tolerability of dPEP itself, not a blinded, potentially placebo version. Second, an open-label design is likely to result in greater (and more realistic) adherence—in placebo-controlled trials of PrEP among African women, adherence was very low, and subsequent qualitative studies found concerns about receiving a placebo as a principal reason, with greater adherence in recent open-label studies [59–62].

The potential for inducing drug-resistant STIs and other important colonizing pathogens is an important concern for dPEP. On one hand, antibiotic resistance to tetracycline-class drugs is selected relatively easily in some bacteria (e.g*.*, *N. gonorrhoeae*); on the other hand, resistance for *N. gonorrhoeae* is common globally, making additional resistance selection unlikely; other organisms (e.g., *C. trachomatis*) have never demonstrated resistance. Syphilis (*Treponema pallidum*) is at risk of developing tetracycline resistance; however, we anticipate very low rates (< 2%) of syphilis in this population, and thus, the risk of developing resistance should be low. Moreover, if dPEP is successful in preventing STIs, resistance may not occur, as selection should only happen if infection is established, and replication occurs despite selective antibiotic pressure. Overall, we feel there are both important knowns and unknowns that define equipoise for this question.

Investigating the effectiveness benefit of dPEP to reduce STI incidence among Kenyan women who are taking HIV PrEP, while also exploring potential risks and cost-effectiveness associated with dPEP, will hopefully produce sufficient results to serve as a resource to policy makers and organizations working in this field to inform decision making.

## Trial status

This publication was written regarding protocol version 5.0 from September 30, 2020. Recruitment began on February 2, 2020, and is expected to continue until November 2021 with all enrolled participants followed quarterly for 12 months.

## Data Availability

Investigators have full access to the final dataset, and any data required to support the protocol can be supplied on request.

## Abbreviations

AE: Adverse event
AMR: Antimicrobial resistance
ART: Antiretroviral therapy
DAIDS: Division of AIDS
DALY: Disability-adjusted life year
dPEP: Doxycycline post-exposure prophylaxis
DSMB: Data safety and monitoring board
ECCT: Expert Committee on Clinical Trials
FTC: Emtricitabine
GEE: Generalized estimating equations
HAL: Hair analytical laboratory
HIV: Human immunodeficiency virus
ICER: Incremental cost-effectiveness ratio
IDI: In-depth interviews
IPV: Intimate partner violence
IRB: Institutional Review Board
KEMRI: Kenya Medical Research Institute
LARC: Long-acting reversible contraceptive
LMP: Last menstrual period
MOH: Ministry of Health
MSM: Men who have sex with men
NAAT: Nucleic acid amplification test
PCR: Polymerase chain reaction
PEP: Post-exposure prophylaxis
PID: Pelvic inflammatory disease
PPB: Pharmacy and Poison Board
PrEP: Pre-exposure prophylaxis
RPR: Rapid plasma reagin
SAE: Serious adverse event
SERU: Scientific Ethics Review Unit
SMS: Short message service
STIs: Sexually transmitted infections
TDF: Tenofovir disoproxil fumarate
UCSF: University of California San Francisco
UW: University of Washington
